# The Role of Caregivers in Supporting Personal Recovery in Youth with Mental Health Concerns

**DOI:** 10.3390/children12060787

**Published:** 2025-06-17

**Authors:** Denise B. McKern, Govind Krishnamoorthy, Vicki C. Dallinger, Diane Heart, Darryl Maybery

**Affiliations:** 1 School of Psychology and Wellbeing, University of Southern Queensland (UniSQ), Toowoomba, QLD 4350, Australia; denise_mckern@outlook.com (D.B.M.); govind.krishnamoorthy@unisq.edu.au (G.K.); diane.heart@unisq.edu.au (D.H.); 2 Manna Institute, Center for Health Research, University of Southern Queensland (UniSQ), Toowoomba, QLD 4350, Australia; 3 School of Rural Health, Monash University, Melbourne, VIC 3800, Australia; darryl.maybery@monash.edu

**Keywords:** caregiver, CHIME framework, mental health, recovery-oriented care, youth recovery

## Abstract

**Background/Objectives:** Mental disorders that emerge during adolescence frequently extend into adulthood, predicting poor academic and employment outcomes and heavy societal burdens. Novel efforts to improve youth mental health have transitioned from clinical recovery, typically focused on a cure, to a strength-based approach to wellbeing in supporting youth within mental health services. Mental health scholars have appealed for interventions to adopt an ecological system of care approach that integrates the principal caregivers in a young person’s life. Despite preliminary literature indicating the importance of caregivers, little research has focused on the caregiver’s role in supporting personal recovery in youth. **Methods**: This study sought to understand the role of caregivers in youth recovery by employing a qualitative design to inductively analyze the narratives from nine semi-structured interviews with caregivers. Additionally, deductive analysis explored the core five underpinnings of personal recovery connectedness, hope, identity, meaning, and empowerment (CHIME). **Results**: A thematic analysis of the literature identified five themes: providing unconditional love and positive regard; encouraging connection with peers; co-creating a sense of purpose, meaning, and hope; supporting assertiveness and advocacy; and promoting strength and opportunity for mastery aligning with the CHIME framework. The findings will allow health services to understand caregivers’ roles better, thus providing information to guide recovery-oriented and family-centered care.

## 1. Introduction

There are serious global concerns about the recent growth of mental health disorders in youth aged 15 to 24 years. Mental health is considered a “state of wellbeing in which an individual realizes [their] abilities, can cope with the normal stresses of life, can work productively, and can make a contribution to [their] community” [1]. Mental disorders are characterized by clinically significant disturbances in cognitions, emotional regulations, or behaviors and are usually accompanied by impairment of distress [1]. Alarmingly, epidemiological studies have highlighted that more than 75% of all mental disorders develop before the age of 25 and 50% before the age of 15 [2,3,4]. Further, a current report on mental health and wellbeing among youth aged 16 to 24 found that two in five (39.6%) had a mental disorder for 12 months or more [5]. This translates to almost half (46.6%) of all female youth and nearly one-third (31.2%) of all male youth [6]. These statistics are concerning because adolescent development is distinguished by multiple social-emotional and physiological transitions integral to identity formation and autonomy [7,8]. Interference during this stage of development can increase the risk of permeating mental health concerns into adulthood [9,10].

To address mental health and wellbeing among youth, global mental health services have begun a paradigm shift towards recovery-based programs as best practice [11]. Personal recovery is defined as a journey of growth, whereby individuals live meaningful, hopeful, and rewarding lives, with or without the ongoing presence of mental health symptoms [12]. Clinical recovery models, such as the CHIME framework, have been the dominant approach to treating mental health disorders using recovery-oriented and family-centered care [11].

Amongst the first authors to distinguish the key differences between youth and adult populations was Simonds and colleagues [13]. These authors explored the lived experiences of adolescents and caregivers (mothers) in the UK utilizing recovery-oriented mental health services. Their findings proposed three components to youth recovery: loss of self, renegotiating the self, and anticipation of future self. Interestingly, the first two components saw consistency with adult literature, while the third component, the anticipation of future self, suggested that youth may have difficulty conceptualizing their future selves [13]. This indicates a maturational or developmental discrepancy between the youth and adult populations. However, the methodological limitations of this study include poor uptake (6% youth, 8% mothers) questioning the relationship between participation and destabilization fears associated with jeopardizing recovery progress. The potential for sampling bias implies the need for further investigation into youth recovery [13].

**Figure 1:**
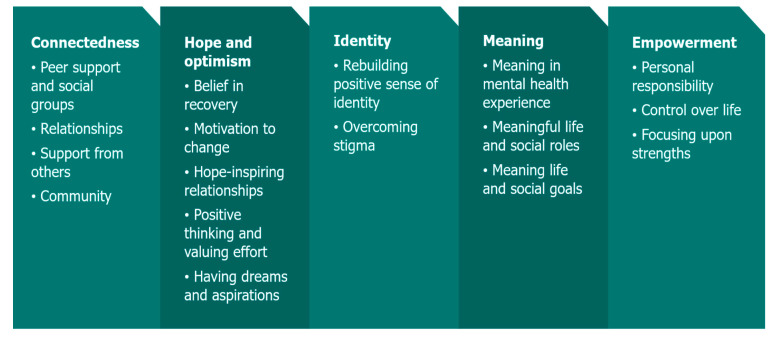
The CHIME framework of personal recovery [14].

A burgeoning body of research has supported the CHIME framework proposed by Slade and colleagues as seen in Figure 1 [14]. CHIME is an acronym for a framework that describes the recovery-related processes and domains: connectedness, hope, identity, meaning, and empowerment [14]. The CHIME framework [14] has undergone rigorous research validation and endorsement, revealing a comprehensive and preeminent conceptual framework for mental health recovery [11,13,15,16,17]. It has been integrated into adult mental health policy reform and practice worldwide, with more recent preliminary findings demonstrating efficacy for youth [18,19].

Dallinger et al. [18] consulted with youth, caregivers, and stakeholders to understand the applicability of the CHIME framework to youth mental health. The thematic analysis revealed that, similar to adults, youth recovery was characterized as a unique, nonlinear, and uncertain journey [14,20,21]. However, unlike adult recovery processes, youth recovery was found to relate to two processes: restoration and resilience. Restorative processes involve dealing with adversity and risk factors, reconnecting with support systems, and building acceptance. Resilience processes aim to bolster the youth’s strengths and protective factors, develop new support networks, and teach self-regulation and advocacy skills [18,22]. Their research underscores the importance of the ecological context in youth recovery [21,23], suggesting interventions should target both the individual’s processes and the opportunities and barriers within their ecological context [22]. Mental health scholars have appealed for interventions that integrate caregivers into all relevant youth care settings.

Caregivers have been found to play a central role in the systems of care model, often facilitating and encouraging access to appropriate treatment whilst being valuable sources for accurate assessment and outcomes monitoring [23]. A carer is somebody “…providing unpaid care to…someone…who has received, is receiving, or is seeking, treatment and support from…health services.” [24]. Policies note the importance of the mental health carer role [25,26,27,28,29]. In 2020, just over 20% of people in the USA were carers, with 35% caring for someone with mental health concerns [30]. Australia has similar numbers, and carers provide an average of 40 h per week of care [31], while in the UK, approximately five million carers provide between 9 and 50 h of weekly care [32]. Sometimes referred to as a critical component in the Triangle of Care [33], carers can provide navigation and communication support to the youth to assist them in gaining the required services within often complex healthcare services [30] and maintain wellbeing.

However, despite preliminary literature indicating the importance of caregivers, there remains a knowledge gap associated with the role of caregivers in youth recovery. Much of the research related to caregivers in youth mental health has focused on caregiver support needs [34,35] and outcomes from intervention [36,37] and particularly on how the key components of personal recovery (e.g., CHIME) might relate to carers and those they support. Understanding the experience and needs of caregivers is essential. The current study seeks to capture the richness of the real-world perspectives of caregivers and their role, as well as the needs associated with these experiences, by utilizing qualitative methodology with its exploratory disposition. As Creswell and Creswell [38] described, the qualitative approach is a fitting application in social exertions, providing a greater understanding of how caregivers experience problems. As caregiver perceptions are subjective and context-bound, the exploratory approach will capture the unique role of caregivers, which is ideal for generating information suitable to inaugurate theories and conceptual frameworks. A modest amount of emerging literature has found the applicability of the CHIME framework [14] to youth mental health recovery [16,18,19], but little is still known about its utility to caregivers. This research aims to address this gap in the study.

## 2. Materials and Methods

### 2.1. Research Design Overview

The current study utilized a qualitative semi-structured interview methodology to explore the lived experiences of caregivers, highlighting their roles in youth mental health recovery. It did this by inductively analyzing the narratives from nine semi-structured interviews with caregivers and generating themes utilizing thematic analysis [39]. Subsequently, deductive analysis explored the core underpinnings of personal recovery under the acronym CHIME to recognize if the themes developed through the narratives aligned within the CHIME framework. This contemporary qualitative method, which seeks to capture the richness and meaning of real-world experiences [38], is considered the most introspective in exploring caregivers’ personal lived experiences and perspectives [40].

Moreover, data was analyzed using reflective thematic analysis (TA), applying a critical realist ontology. This theoretical lens, which has expanded in popularity over the past several decades, offers an inclusive, non-reductionist approach to personal mental health recovery [41,42,43]. Materialized from the rivaling positivist and constructionist paradigms, it uses elements of both approaches to vindicate that ontology (what we know) is not moderated by epistemology (how we know; [41,43]). The postmodernist perspective can be applied to the objective, critical division of the ontology, which relates to the deductive conceptual CHIME frameworks of recovery [14,44]. Contrarily, the realist aspect constitutes the inductive approach, attributed to caregivers’ subjective, lived experiences in youth mental health. Bought together, critical realism posits that reality is assessed through both contextual influences and subjective perspectives [40,43]. Reflective TA can align with critical realism as a flexible method whilst encapsulating social interactional factors central to symbolic interactionism [45]. This will offer greater profundity, allowing for the consideration of a system to be applied to the understanding of youth mental health recovery whilst linking the theoretical and methodological frameworks.

### 2.2. Recruitment and Participants

Ethics approval was granted by the University Higher Research Ethics Committee as part of a more extensive research program. As the research aimed to explore the lived experiences associated with the roles and needs of caregivers in youth mental health recovery, a purposive sample was required. Purposive sampling involves recruiting participants who can provide rich and detailed data to enhance understanding of the research question [39,46]. Thus, the inclusion criteria comprised parents who are the primary care providers of youth aged 12–24 years who are or have utilized mental health services.

Participants were recruited and interviewed by a chief researcher as a component of a more extensive study. The larger study’s broad scope concerned youth, mental health professionals, and caregiver perspectives to explore the conceptualization of youth recovery and translation to digital platforms. The study participants were recruited through personal communication, email, and flyer advertisement within local mental health networks in Queensland and consumer advocacy groups. Potential participants expressed interest by contacting the principal researcher, who then provided an information pack comprising a project information sheet and participant consent form, an interview question sheet guide, and a demographic form. Every inquiry returned signed consent forms and met the inclusion criteria, and no participants were excluded from the study. Participation was voluntary, and participants were eligible to receive remuneration through a retail voucher ranging from $20 to $100 via a random draw. Confidentiality was maintained through data deidentification and the use of pseudonyms.

### 2.3. Participants

An adequate sample size consisting of 9 female caregivers was interviewed as shown in Table 1. Braun and Clark [39] deem this a suitable sample size to capture the insights and depth of information using semi-structured interviews. Participants held a mean of 45 years, with affiliated youth showing a mean of 4.9 years engaged with mental health services. All participants resided within the state of Queensland, Australia. The total number of participants was chosen based on data saturation. Braun and Clark [39] define saturation as gathering enough information to adequately understand the topic or where no new knowledge is being obtained.

**Table 1:** Participant demographic information.

Caregiver	Age	Gender	Time in MH	Location	Education
1	46	Female	9 yearrs	Metro	Post-Graduate
2	49	Female	2 years	Rural	Year 12
3	40	Female	4 years	Regional	Diploma
4	53	Female	4 years	Metro	Bach Degree
5	44	Female	4 years	Metro	Bach Degree
6	45	Female	2.5 years	Metro	Certificate111
7	50	Female	7.5 years	Metro	Year 11
8	44	Female	5 years	Rural	Assoc Dip
9	42	Female	2 years	Regional	Bach Degree

Note. Time in MH = The number of years that the youth has been in mental health services. Metro = Metropolitan; Education = Highest level of education undertaken; Bach Degree = bachelor’s degree; Assoc Dip = Associate Diploma.

### 2.4. Data Collection

Data was collected through semi-structured interviews. Interview questions are available in the supplementary materials. All interviews were digitally recorded and began by requesting demographic information followed by several pre-determined, open-ended questions applied to guide the interview. In this manner, semi-structured interviews promote spontaneous and unplanned questions, drawing out participant responses that enhance understanding of the research topic’s essence [39]. Interview durations ranged from 35 to 65 min, depending on participants’ engagement and the depth of responses. All interviews were transcribed using the Panopto automatic speech recognition program. All data was reviewed for accuracy and stored according to university data security procedures.

### 2.5. Data Analysis

Thematic analysis (TA) was used to analyze data using Nvivo version 12, allowing for a flexible and comprehensive explorative research method [39]. TA provides a systemic approach to analyzing and identifying patterns known as themes in datasets. During the theme development stage, an inductive approach was employed, ensuring that the researcher was guided by the content rather than a pre-existing theoretical lens [38]. Data was coded by two of the authors, and themes were developed through an iterative process.

## 3. Results

The study aimed to explore recovery-oriented caregiving- understanding the role of caregivers in supporting personal recovery amongst youth with mental health concerns. The analysis identified five themes: (1) providing unconditional love and positive regard; (2) encouraging connections with peers; (3) co-creating a sense of purpose, meaning, and hope; (4) supporting assertiveness and advocacy; and (5) promoting strength and opportunities for mastery. Table 2 displays the themes and illustrative quotes. Verbatim extracts from the transcripts have been presented, with ellipses […] to indicate where data has been omitted for succinctness. These have only been utilized where the omission does not alter the interpretation.

### 3.1. Theme 1: Providing Unconditional Love and Positive Regard

Unconditional positive regard is a concept coined by humanistic psychologist Carl Rogers. It is defined as an attitude of caring and acceptance towards other individuals, regardless of their behaviors and actions [47]. Unconditional love is affection without limitations or conditions; it has no bounds and is unchanging [48]. This study’s caregivers strongly endorsed that no matter what challenges life throws at them, their unconditional love will prevail, reinforcing the parent-child connection and bond.

Offering unconditional love and acceptance can be a challenge for caregivers as the process of supporting youth can, at times, be stressful and frustrating. Simultaneously, the evaluations of others and the dominant discourses of youth mental health can profoundly affect them, rendering it more challenging to muster the compassion felt at the earlier stages of illness. Further, participants described how they were often subjected to stigmatizing throw-away comments, leaving them feeling blamed and responsible for their young person’s mental challenges, as described by Caregiver 4:

“The general sense from everyone I speak to is that their normal network of friends and family is difficult to talk to because they don’t get it. [...] But it’s not because they’re not getting the complexity [...] it is an illness you know their thinking is behavioural.”

These judgments are intimately connected to participants feeling inferior and responsible for their young person’s mental health decline. A wealth of literature has suggested that parents, more specifically mothers, are regularly blamed for the etiology of their child, with frequent reference being made to ineffective parenting and lack of discipline [49,50,51,52].

Coping with the daily psychopathology of the young person, accompanied by mood disturbances and socially inappropriate and uncooperative behaviors, can be incredibly distressing. Caregivers may find it challenging to maintain empathy, especially when experiencing self-doubt, as reinforced by Caregiver 6:

“It makes you sort of question your parenting, and in some ways, it changes how you think people perceive you as a parent, too.”

Caregivers often expressed that they adopted stoic and philosophical attitudes to look beyond disparagingly dominant discourses. They expressed that they had placed their energy behind what they could control: accepting their circumstances and building their young person’s self-confidence and self-esteem through love, acceptance, and understanding. Caregiver 1 summed up thoughtfully:

“Being a little lighthouse […], you know, shining the light for her.”

Many caregivers applied praise and extended compliments in the hope that youth would feel better about themselves, which in turn would reflect upon enhancing recovery. This was reinforced by Caregiver 6, who described:

“Building her self-confidence through compliments and through other ways about understanding her perception of self as well.”

Caregivers believed showing support by making themselves available to the young person was the best way to display unconditional love.

### 3.2. Theme 2: Encouraging Connection with Peers

The theme of encouraging connection with peers described the need to promote a sense of connection to aid youth in their recovery journey. Connection was described as breaking the isolation pattern to help youth feel a sense of belonging. Fittingly, caregivers expressed that their role in recovery often saw them instigate youth friendships, recognizing that the young person wanted to connect but frequently did not know how:

“I have to pretty much instigate any catchups with his peers. He never instigates anything […]. I think sometimes he wants to connect but doesn’t know how to”.

(Caregiver 4)

Providing a sense of connectedness was perceived as imperative to recovery. However, caregivers were often met with resistance when they endeavored to open dialogue or initiate connections with peers, as highlighted by Caregiver 3, who expressed:

“She didn’t want to connect […] she was pretty resistant to connecting. She wanted to know that she hadn’t been forgotten.”

These findings echo literature centered on the concept that belonging emerges from motivation. The belonging-motivation model by Leary and Kelly [53] proposed that weak motivation may predict psychological difficulties. A lack of motivation may develop from repeated rejections, thus hampering a person’s basic psychological needs for relatedness [54]. This lack of motivation is assumed to result in a learned helplessness response, which reduces motivation to belong [53].

However, Caregiver 4 believed that for her son, the reluctance to develop peer relationships was geared to feelings of inadequacy and trust, which fundamentally stemmed from challenges attributed to social anxiety:

“Well, I think he feels like he is an outsider, and he is different to his peers. He feels like he connects more to adults than his peer group. For him, it’s been difficult to make connections because of his lack of trust and his level of anxiety in social settings, and it’s also been difficult for him to branch out away from me […] because he didn’t want to separate from me.”

Research has continually demonstrated that individuals who feel connected are better able to cope, less prone to anxiety and depression, and have overall better physical health [55,56]. Connecting to peers, family, and the community is essential for young people. Within these contexts, youth can define their identities and seek experiences that enhance their sense of self and emerging adult capabilities [57]. It is also suggested that youth who are better connected with their families and schools have fewer occurrences of mental illness [58]. This is consistent with CHIME’s framework of connectedness [14].

### 3.3. Theme 3: Co-Creating a Sense of Purpose, Meaning and Hope

The theme of co-creating a sense of purpose, meaning, and hope evoked significant dialogue. All caregivers subscribed that purpose and meaning were central to supporting a young person’s recovery. First, caregivers described that a large part of their recovery role was to assure and (to varying degrees) persuade their youth that their lives have a purpose. As a caregiver reflected:

“I think he probably needs to know that he has a purpose. That there’s a purpose to being alive and for that to be part of the therapy goal”.

(Caregiver 4)

Often, this was achieved by aiding their young person to cope with insecurities to improve their understanding of themselves and building a connection to key figures. All caregivers spoke about how their role in recovery involved co-creating goals with the youth, allowing the youth to focus less on symptomatology and more on interests, preferred activities that provide them with a sense of self-worth and happiness. Caregiver 3 reflected:

“Having an idea about what his future or some of it looks like. Actually, having some future goals.”

These findings have emphasized the relevance of the CHIME framework to meaning in youth recovery [14]. This is also consistent with the findings of Naughton et al. [16], who reported that meaning in life for a young person involves normative social connections, goals, and a positive outlook.

However, some division was recognized among caregivers’ views towards adjusting to goals and life beyond mental health. These caregivers expressed that they needed to reinforce self-compassion and self-worth, as highlighted here:

“That she’s gained a load of self-worth and feeling good about herself and happiness with what she’s doing in her life. Purpose, and a bit of direction”.

(Caregiver 1)

This proposed phase of recovery is conceptually similar to Andresen et al.’s [59] moratorium (a period of identity confusion, hopelessness, and social withdrawal) and Spaniol et al.’s [60] overwhelmed by the disability (a phase of confusion, fearfulness, and disconnection from self and others). At this stage, the role of a caregiver may be more in line with the conceptualizations of Naughton et al. [19], re-engaging their young person in the usual day-to-day activities rather than reforming or re-building their lives.

This newfound focus advanced a more progressive stage of recovery, enabling caregivers to regain more control to co-explore meaning as unique and multifaceted. Where purpose personified establishing routines and larger goals, as well as spending time with pets, friends, family, and outdoor activities. This was highlighted by Caregiver 4:

“Gaining control back ... just re-establishing routine and helping him to know that he can do it. Like that sense of accomplishment and knowing that he’s supported by a small community, and everyone’s connected.”

This phase of recovery represents a greater sense of social re-engagement. It resembles the conceptual similarities to Andresen et al.’s [59] awareness stage (a realization that a different self is possible) and Spaniol et al.’s [60] struggling with the disability phase (an increased knowledge of the problem and a growing of self-confidence, yet still holding onto some fear of destabilization).

The sentiment of hope is another facet of the CHIME framework, which considers establishing close bonds and relationships an integral part of this process [14]. Most caregivers explained that youth needed to shift their thinking to open themselves to feeling hopeful. This entailed altering thoughts and feelings of despair to a more optimistic outlook of their future, occupying the belief that life will improve despite the limitations of the current mental health challenges. Most caregivers explained that promoting hopeful mindsets meant fortifying close parental connections with a strong emphasis on mentoring and persistent reassurance.

However, most participants drew light on the notion that to instill hope in youth, caregivers needed to portray a sense of hopefulness themselves. Hope theory is the closest theoretical link that may illuminate how caregivers draw hope to themselves and their roles [61]. Hope theory contends that hope is a product of three central aspects: goals, pathways, and agency, which are responsible for aiding or diminishing one’s hope. This theory provides the conception that to be hopeful, one must garner resources and insight to feel empowered to achieve desired goals. In this way, caregivers’ roles could see them set personal goals for themselves and their youth and develop strategies to help achieve these goals. However, to our knowledge, this concept is unexplored in caregiving and youth mental health literature, providing a reason to explore these findings further.

### 3.4. Theme 4: Supporting Assertiveness and Advocacy

When caregivers were asked what they thought was important in the young person’s recovery journey, the collective response emphasized facilitating confidence and self-esteem. As stated by Caregiver 6, coping skills and capacity building were considered essential elements in youth recovery:

“It’s really sort of about helping them to build the skills that they need to manage those sorts of situations that can pop up.”

Further, several caregivers explained that their role in recovery was to encourage the young person to be involved and make choices and independent decisions relating to their treatment, as recalled by the following account:

“Being involved in their treatment is such a good thing, you know. Basically, they’re adults, aren’t they? So, for her to have that sense that she is to a degree in charge of her own recovery is a great thing”.

(Caregiver 2)

However, a commonly shared concern among caregivers was that although they understood that balancing autonomy and belonging is a normative developmental challenge, they were often confused about where to draw the line between typical adolescent behavior and the precursors of mental illness. This was fortified by Caregiver 6:

“Sometimes it’s just like knowing what’s adolescent behaviour, and what may be a sign of something not being quite right.”

Furthermore, expanding on systems approaches, many caregivers expressed that they would often encourage their young person to establish self-directed connections with their service providers and practitioners to promote advocacy and assertiveness, as featured here:

“Teaching her that her psychologist is her safe space. So, [youth] goes into all of those sessions by herself. That’s her space. That’s her space to share and have really good discussions, to speak up for herself”.

(Caregiver 9)

These findings highlight youth recovery features corresponding to the CHIME empowerment framework [14]. Consistent with Naughton et al. [16], the caregivers emphasized that their roles in endorsing self-efficacy with mental health services fostered hope and optimism for the youth and allowed them to recognize their strengths and abilities to deal with and overcome challenges. This sense of empowerment apportions the youth’s active role in managing their mental health, allowing them to acquire autonomy over their functioning and wellbeing [19].

The dominant understanding amongst caregivers was that their role in supporting youth was continually changing, which caused a need to adapt their interactions accordingly to facilitate both moments of growth and regression. However, the emphasis was predominantly geared towards gradually stepping away and imparting the youth a greater sense of self-management and self-regulation in their recovery. One caregiver commented:

“I’m not going to hold you with me. You have got to do this on your own. You have to manage your illness; you have to manage your own. This is your journey, not my journey […] it’s your choice”.

(Caregiver 6)

Most caregivers felt that they could perform a better role in caregiving if mental health services better supported them, as expressed by Caregiver 7:

“Parents [need] to have that basic mental health training. Because sometimes I think to myself, when (young person) asks me stuff, I don’t know if I’m actually making the situation worse or better because I don’t know if I’m responding correctly.”

Caregivers are often the primary support system that remains stable throughout treatment adjustments [62], and they play a prominent role in steering their young person’s mental health recovery.

### 3.5. Theme 5: Promoting Strength and Opportunities for Mastery

The theme promoting strength and opportunities for mastery was dominated by caregivers articulating a solid emphasis on resilience. Resilience is the process and outcome of adjusting and adapting to challenging experiences through mental, emotional, and behavioral flexibility [63]. Caregivers promoted reliance building by motivating youth to take charge and apportion the responsibility of managing their choices. Caregivers often endeavored to focus on the youth’s strengths and experiences as an opportunity to recognize and influence their capabilities, valuing their abilities to aid recovery as voiced here:

“Celebrating the things that she hasn’t been able to do before, and I say to her, you need to celebrate those little achievements that you made. Celebrating how well she is doing, you know, which is what you and I would do as a normal thing. She needs to acknowledge how well she has done so for her to have that sense that she is, to a degree, in charge of her own recovery is a great thing”.

(Caregiver 2)

However, caregivers also stipulated the importance of honesty, taking care not to underplay the challenges and, at times, unpleasant emotions that accompany the youth recovery journey, as articulated here:

“Being told that she is a strong, resilient young person who can do this thing. […]. Also being really honest and saying, but it’s not going to be comfortable. In fact, I had a conversation with her; she failed her driving test on Monday; it was just all over the place. We had this really good conversation where I said, look, you know, disappointment sucks, hurts, really hurts. But you’ll get through it, and you learn from it”.

(Caregiver 3)

Erikson’s [64] psychosocial developmental theory is of continued relevance and provides a contextual framework to consider a young person’s developmental complexities. Moreover, within the identity and role confusion stage, Erikson [7] coined the phrase ‘identity crisis’, which is linked to the psychological moratorium where teens place their current identity on hold while exploring other identity options [65]. However, when identity formation is compromised by mental illness, promoting strength and resilience can be a genuine struggle for caregivers. This echoes literature suggesting that youth who succumb to the dominant misperceptions of mental illness may develop ‘self-stigma’- a negative internalization and self-appraisal which stems from being stigmatized, often leading to low self-esteem and self-efficacy [66].

## 4. Discussion

The present study is the first to explore a conceptual understanding of the caregiver’s role in supporting personal recovery in youth with mental health concerns. The study found five themes: (1) providing unconditional love and positive regard; (2) encouraging connection with peers; (3) co-creating a sense of purpose, meaning, and hope; (4) supporting assertiveness and advocacy; and (5) promoting strength and opportunities for mastery. These themes supported the CHIME framework [14] of recovery and preceding literature exploring recovery as applied to young people [16,18,23], along with providing important information about the role of carers with young people.

Unconditional love and acceptance were ascertained as the fundamental facet, often regarded as the foundation from which all other elements of recovery could be cultivated. Further, the primary way caregivers influence youth recovery is through their relationship with the youth through connection. Notably, caregivers challenge the self-stigmatizing views of their youth. Kelly and Coughlan [23] also noted that youth recovery lies within the ecological context of complex, hierarchal, interconnected social relationships. They stated that recovery for young people requires connection and acceptance. Connection allows youth to experience hope, acceptance, positivity, and normality. These are all essential elements for youth in developing resilience and gaining a sense of empowerment and control over their mental health journey.

Caregivers scaffold the risk management and treatment offered by mental health professionals. They also provide support that helps youth function across settings—friends, school, and mental health services. They challenge the stigmatizing views of stakeholders in these settings towards their youth and themselves as a family. Despite changing attitudes towards mental health, stigmatizing views permeate through the public and even youth-serving systems. Further investigation into the role of caregivers in youth mental health is necessary to improve understanding of the role of ecological supports surrounding youth recovery.

Caregivers need to be attuned and flexible in how they respond to youth. Practical implications of this research highlight that caregivers need education, training, and support to reduce and even avoid succumbing to stigmatizing views of mental health and diagnosis that can lead to becoming risk-aversive and overly protective. Education and training can offer knowledge that may alleviate the guilt associated with the sense of having caused these mental health difficulties for young people. Health systems adopting a recovery-oriented approach to care could further support clients by offering such training and education to caregivers of youth engaged with services. For caregivers, the challenges of maintaining strong relationships include balancing youths’ needs for autonomy and independence whilst supporting themselves to manage their competing demands and engaging in self-care. Dyadic coping skills may offer support to both parents and youth in managing mental health and connection. Health services supporting young people may also consider supporting caregivers and youth with developing dyadic coping skills as a critical aspect of recovery-oriented care.

### Limitations and Future Research

There were several limitations within this research. Although efforts were made to recruit broadly, all caregivers were affiliated with community-based programs that provided services to young persons with chronic mental health, resulting in limitations with sampling. Presumably, the caregiver support role would elicit unique challenges and insights when no community support has been sought and when youth experience milder mental health challenges. These contextual elements, along with socioeconomic status, cultural background, and diagnosis, are noteworthy elements allowing for richer interpretation, especially given the study’s critical realism stance.

Further, efforts were made to recruit an equal representation of male and female caregivers. However, the perspective of mothers was solely represented. This means findings are limited to the meanings of mothers, limiting the generalisability of the caregiving role broadly across the parental domains. As mothers are traditionally viewed as nurturers and caretakers, the themes generated, like unconditional love and positive regard, could be more reflective of their traditional roles. Despite the increased engagement of men in parenting, paternal caregiving representations in youth recovery have, to our knowledge, not been investigated.

Despite the empirical strength of the CHIME framework [14], applying this model deductively as a theoretical basis of this current study may have potentially limited the perspectives shared by caregivers. Youth recovery is an emerging construct and, until recently, lacked theoretical conceptualization. Future research exploring the conceptualization of youth recovery outside the CHIME framework [14] could offer alternative views and understanding of this construct and insight into the role of caregivers in youth recovery.

Caregivers have been identified as a critical component of youth mental health recovery through this and previous research. Future research could build on these findings and generate a conceptual framework of the roles and needs of parents in supporting youth recovery. Such a framework would provide a guide to recovery-oriented and family-centered care and help to address the growing concerns regarding mental ill health in young people.

## Figures and Tables

**Table 2:** Summary of findings generated through thematic analysis.

Main Theme	Illustrative Quote	Corresponding CHIME Acronym
Providing unconditional love and positive regard	“Unconditional love from family and friends. For us to accept who they are and where they’re at” (Caregiver 4)	Connection
Encouraging connection with peers	“... and I say to him, have you messaged your friends? And he’s like “Nah.” And I’m like, do you think you should?” (Caregiver 4)	Connection
Co-creating a sense of purpose, meaning, and hope	“Having an idea about what his future looks like […] future goals […] something to work towards [...] that he can hold onto as you are doing day-to-day” (Caregiver 3)	Meaning Connection Hope
Supporting assertiveness and advocacy	“I think that’s a big thing for her … being able to make choices … what she feels works and what doesn’t work” (Caregiver 6)	Empowerment Connection
Promoting strength and opportunities for mastery	“I think she isn’t a fragile small child that has to be cossetted. Being told that she is a strong, resilient young person who can do this” (Caregiver 2)	Empowerment Identity Connection

## Data Availability

The data analyzed during the current study is not publicly available due to client confidentiality but is available from the corresponding author on reasonable request, although restrictions apply to the availability of the data.

## Supplementary Materials

The following supporting information can be downloaded at: https://www.mdpi.com/article/10.3390/children12060787/s1, Supplemental A: Interview Questions. Supplemental B: Demographic Form.
