# Supplementary material for: The Role of Caregivers in Supporting Personal Recovery in Youth with Mental Health Concerns
Source: Children (Basel). 2025 Jun 17;12(6):787. doi: 10.3390/children12060787 (PMC12192533; doi:10.3390/children12060787)
Supplement: Supplementary file 1 [file children-12-00787-s001.zip › children-3621277-supplementary.pdf]

The Role of Caregivers in Supporting Personal Recovery in Youth with Mental Health Concerns

Supplemental A: Interview Questions

Parent/Carer Questions

The questions below will help us understand parent and carer understanding of adolescent recovery, and how apps and digital health applications can help. Some questions are referring to your young person, and some are referring to your own experience. Some questions have options that allow you to choose how they respond. Please consider all questions and provide answers on the below sheet.

Question 1

What Apps and internet tools are you using to help your adolescent with their mental health concerns and/or difficulties?

When are you (i.e. the parent / carer) /they (i.e. adolescent) using them?

____
____
____
____
____
____
____
____

How do they help your young person?

____
____
____
____

Question 2

What do you think needs to happen to help your young person feel more connected to other people and services?

What would help you as a Parent/Carer to connect to other people and services?

What would be the benefits of connecting to other Parents/Carers within the mental health service?

Question 3

What do you think needs to happen to help your young person feel more hopeful and optimistic about their future?

Or

If you woke up tomorrow and your young person was feeling better what would have happened?

Question 4

During adolescence, young people begin to explore their independence and develop a sense of self. Connections, beliefs, and goals may form to help our young people discover who they are and what is important to them.

What do you know about how your young person identifies themselves? What makes them who they are?

What has happened within your young person's journey that has impacted your identity as a Parent/Carer and as a person?

Question 5

What do you think is important to your adolescent and gives them a sense of purpose?

What is important to you and gives you a sense of purpose?

Question 6

What do you think needs to happen to help your young person feel more in control of getting better?

What was helpful for you during periods of uncertainty and/or difficulty during your young person's journey?

Question 7

If there was a website, app or online program to support you and your child with their recovery, what information would it contain? What would it look like? How would you use it?

Supplemental B: Demographic Form

Age____

Gender____

Time with Services______________________________

Location_______________________________________

Highest Level of Education________________________
